# Thyroid metastases from breast cancer: a case report and brief literature review

**DOI:** 10.3389/fonc.2025.1614376

**Published:** 2025-09-11

**Authors:** Siqi He, Jicheng Li, Donglai Wang, Qiaohong Nong, Guangxin Li, Ying Yin, Xiaoling Liu

**Affiliations:** ^1^ Department of Breast and Thyroid Surgery, Peking University Shenzhen Hospital, Shenzhen, China; ^2^ Shenzhen University Medical School, Shenzhen, China; ^3^ PKU-Shenzhen Clinical Institute of Shantou University Medical College, Shantou, China; ^4^ Department of Oncology, Peking University Shenzhen Hospital, Shenzhen, China; ^5^ Department of Pathology, Peking University Shenzhen Hospital, Shenzhen, China

**Keywords:** thyroid metastases, breast cancer, immunohistochemistry, diagnosis, treatment

## Abstract

Thyroid metastasis from breast cancer is a rare occurrence and often indicates a poor prognosis. We report the case of a young female patient with thyroid metastasis from breast cancer after being diagnosed with the Graves’ disease, and review the clinical characteristics and diagnostic approach of thyroid metastases. The mechanism may be associated with altered microenvironment induced by the Graves’ disease and Hashimoto’s thyroiditis. Thyroid function and abnormal imaging examination should be paid attention during breast cancer patients’ follow-up. Early identification and individualized treatment of thyroid metastasis may contribute to prolonged survival and improved quality of life.

## 1 Introduction

The most common distant metastatic sites of breast cancer are the bone, liver, brain and lung, while metastasis to the thyroid gland is rare (1). Thyroid metastases originating from non-thyroidal malignancies are relatively uncommon and are generally associated with poor prognosis (2). The principal causes of hyperthyroidism include Grave’s disease and toxic nodular goiter (3). In certain cases, patients with thyroid metastases may present with hyperthyroidism due to tumor-induced destruction of thyroid follicles (4). However, there are few cases of primary hyperthyroidism occurring before metastasizing to the thyroid. Herein, we report a case of a patient with early-breast cancer subsequently developed thyroid metastases following treatment for the Graves’ disease.

## 2 Case presentation

A 34-year-old nulliparous woman was diagnosed with right breast cancer in November 2016. She underwent nipple-sparing mastectomy with sentinel lymph node biopsy. Histopathological examination confirmed invasive ductal carcinoma of the right breast, grade 2, without evidence of lymph node metastasis or lymphovascular invasion. Immunohistochemistry (IHC) demonstrated estrogen receptor (ER 80%) and progesterone receptor (PR 5%) positivity, human epidermal growth factor receptor 2 (HER2) negativity, and a high proliferation index (Ki-67 40%), classifying it as Luminal B HER2-negative. The pathological TNM stage was pT1bN0M0. The patient subsequently received six cycles of adjuvant chemotherapy with TC (docetaxel 75mg/m^2^ and cyclophosphamide 1000mg/m^2^) followed by endocrine therapy with tamoxifen (20mg p.o. qd).

The patient was followed regularly according to the planned surveillance schedule, with no evidence of recurrence or distant metastasis. In January 2020, thyroid ultrasonography revealed a 4×3 mm solid hypoechoic nodule in the inferior pole of the right thyroid lobe. Given the small size of the lesion, biopsy was not performed and the patient was advised to continue routine follow-up. Subsequent evaluations demonstrated no significant changes in the size or characteristics of the nodule. In August 2023, the patient reported symptoms of neck swelling, hand tremors, exophthalmos, and weight loss. Thyroid ultrasonography at that time showed diffuse enlargement of the thyroid gland with increased vascularity. Hyperthyroidism was confirmed by thyroid function tests (**Table 1**). The patient was diagnosed with Graves’ disease. The endocrinologist recommended the treatment with methimazole, which resulted in stabilization and normalization of thyroid function (**Table 1**).

**Table 1:** The changes of thyroid function.

Item	Reference range	Aug 2023	Sep 2023	Oct 2023	Nov 2023	Jan 2024	Mar 2024	May 2024	Jul 2024	Oct 2024	Dec 2024	Jan 2025
FT3 (pg/mL)	1.9-4.8	14.47	2.96	7.67	5.23	3.63	2.72	3.28	2.80	2.73	3.45	3.19
FT4 (ng/dL)	0.62-1.24	4.67	0.67	2.76	1.81	1.07	0.62	0.84	1.10	0.94	0.83	0.87
TSH (mIU/L)	0.56-5.91	<0.005	<0.005	<0.005	<0.005	<0.005	0.212	3.146	2.491	3.219	1.847	2.922
TPOAb (IU/mL)	0-9.0	363.3									30.9	27.8
TGAb (IU/mL)	0-4.9	14.6									29.6	24.1
TRAb (IU/L)	0-1.5	>30									1.11	0.94

**Figure 1:**
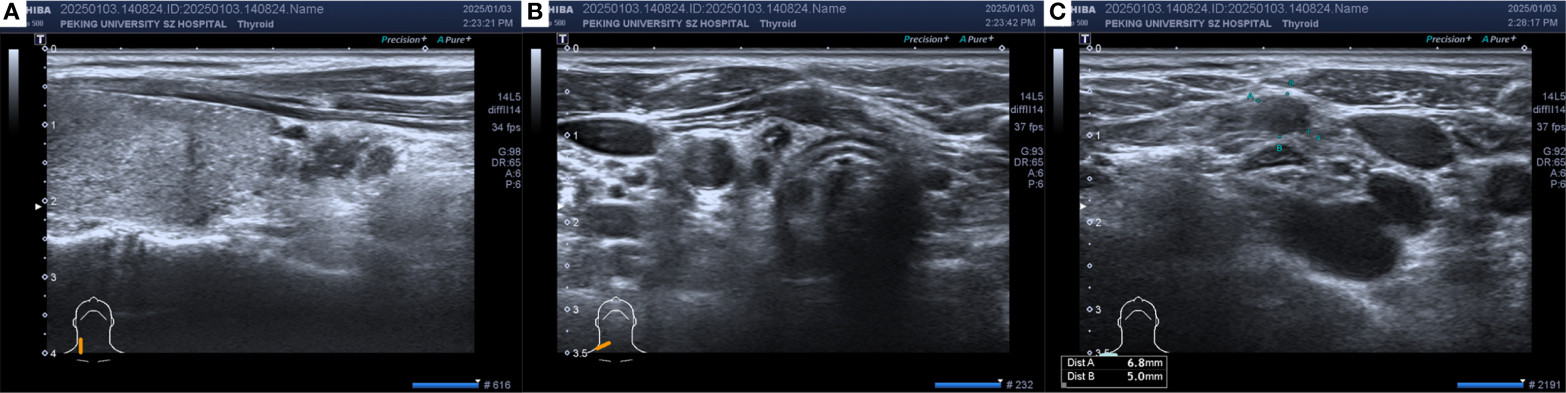
The thyroid ultrasound in December 2024: **(A)** Hypoechoic thyroid nodule located in the right thyroid lobe. **(B)** Enlarged lymph node in the central neck region (Level VI). **(C)** Enlarged lymph node in the right supraclavicular region. Heterogeneous echogenicity in the right thyroid lobe, with multiple scattered punctate hyperechoic foci. A hypoechoic nodule measuring approximately 13×4×8 mm with an irregular shape and punctate hyperechoic foci inside was observed in the lower part of the right lobe. Multiple hypoechoic nodules with irregular shapes, clear boundaries, cortical thickening, and loss of the lymph node hilum structure were found from level IV of the right neck to the right supraclavicular region, with a few hyperechoic foci inside.

**Figure 2:**
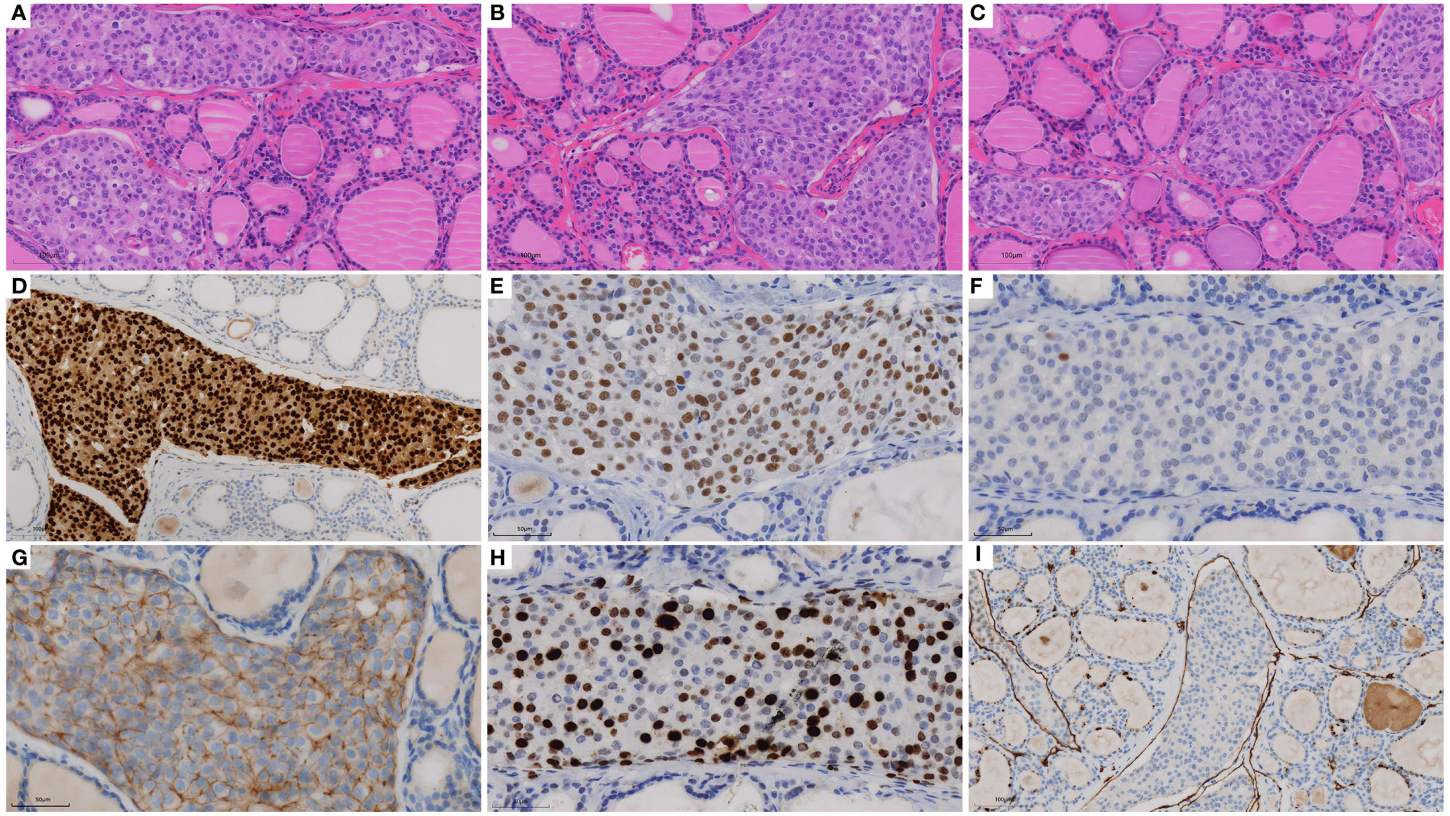
H&E staining and immunohistochemistry of biopsy specimens: **(A-C)** H&E of thyroid (×20), **(D)** GATA3 (×20), **(E)** ER (×40), **(F)** PR (×40), **(G)** HER2 (×40), **(H)** Ki-67 (×40) and **(I)** D2-40 (×20).

In December 2024, thyroid ultrasonography demonstrated diffuse parenchymal changes in the right lobe of the thyroid (**Figure 1A**), accompanied by multiple enlarged lymph nodes in the central and right lateral neck compartments (**Figures 1B, C**). A significant increase in the size of the nodule in the inferior pole of the right thyroid lobe was also noted (**Figure 1A**). Fine-needle aspiration biopsy (FNAB) of the nodule revealed features consistent with papillary thyroid carcinoma, with the possibility of a special histologic subtype. FNAB of the right lateral neck lymph node demonstrated findings suspicious for metastatic carcinoma, although the thyroglobulin (TG) level was undetectable (<0.1 ng/mL). Based on these results, the patient was diagnosed with papillary thyroid carcinoma of the right lobe with lateral lymph node involvement and subsequently underwent total thyroidectomy, bilateral central neck dissection (Level VI), and right radical neck dissection (Level II, III, IV and Vb). Postoperative pathology revealed that the right thyroid lobe was entirely replaced by a tumor, which was confirmed to be metastatic breast cancer (**Figures 2A-C**), with extensive lymphovascular invasion (**Figure 2I**) and lymph node metastases (8/8 in Level VI and 8/31 in Level II, IV and Vb, for a total 16/39). IHC demonstrated positivity for GATA3, ER (60%) and PR (2%), with HER2 negativity and a high proliferation index (Ki-67 70%) (**Figures 2D-H**), findings concordant with the patient’s previous diagnosis of Luminal B, HER2-negative breast cancer. The left thyroid lobe exhibited histological features of Hashimoto’s thyroiditis. Postoperative positron emission tomography/computed tomography (PET/CT) showed no evidence of distant visceral metastases, and serum tumor markers remained within normal reference ranges. The patient was advised to transition to an endocrine therapy regimen consisting of ovarian function suppression (OFS) with an aromatase inhibitor (AI) and a cyclin-dependent kinase 4/6 inhibitor (CDK4/6i), along with adjuvant radiotherapy to the right neck for metastatic breast cancer. Levothyroxine was also prescribed for thyroid hormone replacement. At present, the patient remains in stable condition with no evidence of recurrence or distant metastasis on serial surveillance imaging.

## 3 Discussion

The incidence of thyroid metastases is rare, with the kidney reported as the most common site of origin for primary tumors (5–7). The incidence of thyroid metastases from breast cancer has been reported to range between 7.8 and 13% (7, 8). Routes of metastatic spread to the thyroid include direct invasion, retrograde lymphatic dissemination, and hematogenous spread (9). Breast cancer is generally believed to metastasize to the thyroid primarily via restrograde lymphatic dissemination (9). A recent literature review identified 79 documented cases of breast cancer metastasizing to the thyroid, reported across 46 publications between 1962 and 2022 (10). The predominant histopathological subtype was invasive ductal carcinoma, with hormone receptor-positive tumors being the most common molecular subtype (10). The interval between the diagnosis of the primary tumor and the detection of thyroid metastasis varied widely, ranging from 2 months to 22 years (10).

The thyroid gland is a highly vascularized organ. However, metastasis to the thyroid is rare, which may be attributed to its unique physiological environment. Willis proposed two hypotheses to explain this phenomenon: 1) the rapid blood flow within the thyroid prevents malignant cell adhesion, and 2) the high oxygen saturation and elevated iodine concentration inhibit malignant cell growth (11). Other researchers have suggested that thyroid disorders such as Hashimoto’s thyroiditis and nodular goiter may disrupt the thyroid microenvironment, thereby creating favorable conditions for malignant cell colonization and proliferation (9, 12, 13). In addition, as some patients present with concurrent primary thyroid carcinoma, it has been proposed that primary thyroid cancer may establish a supportive tumor microenvironment that facilitates metastatic tumor growth and contributes to the mechanism of thyroid metastasis (14).

Patients with thyroid metastases often present with clinical manifestations similar to those of primary thyroid carcinoma, including thyroid nodules, hoarseness, and dysphagia caused by compression of adjacent structures such as nerves and esophagus (4, 15). Thyroid function is usually normal. However, cases of both hyperthyroidism and hypothyroidism have been reported (13, 16, 17). Hyperthyroidism may occur when malignant cells disrupt thyroid follicles, leading to uncontrolled release of thyroid hormones (17). Conversely, progressive tumor growth can ultimately impair thyroid function, resulting in hypothyroidism (17). In the present case, thyroid dysfunction was first observed in August 2023, seven years after breast surgery. The patient was diagnosed with the Graves’ disease by the endocrinology department, supported by a markedly elevated TRAb level (3, 18). Concurrently, Hashimoto’s thyroiditis was confirmed by the presence of thyroid autoantibodies and histopathological (18). Her hyperthyroidism was successfully controlled with the treatment of methimazole. This represents a rare case of breast cancer metastasis to the thyroid in a patient with pre-existing the Graves’ disease. Of particular interest, this patient exhibited both Graves’ disease and Hashimoto’s thyroiditis. While Hashimoto’s thyroiditis is typically associated with hypothyroidism, the coexistence of hyperthyroidism suggests an overlapping autoimmune process (19). We hypothesize that the patient’s thyroid microenvironment and vascular dynamics were altered by autoimmune inflammation, thereby facilitating metastatic colonization and growth of breast cancer cells in the thyroid. The existence of autoimmune thyroiditis may have created a permissive microenvironment more susceptible to metastatic implantation and progression. Nonetheless, this hypothesis remains speculative and requires further investigation.

Ultrasound remains the first-line imaging modality for thyroid diseases (20). However, distinguishing metastatic lesions from primary thyroid malignancies using ultrasound alone is challenging (4, 21). Thyroid metastases can be classified into two sonographic patterns: nodular and diffuse. The nodular type is characterized by single or multiple solid hypoechoic lesions with irregular margins and reduced vascularity (22, 23), whereas the diffuse type presents as a hypoechoic lesion involving the entire thyroid gland (22, 23). In the present case, ultrasound demonstrated heterogeneous echogenicity and diffusely scattered microcalcifications in the right lobe, findings consistent with reported cases (13, 21, 24).

FNAB is the preferred diagnostic method for evaluating thyroid lesions and differentiating benign from malignant disease. However, distinguishing primary thyroid carcinoma from thyroid metastases based solely on cytomorphology is challenging and requires IHC for confirmation (5, 25). Primary thyroid carcinomas typically express TG, TTF-1, and PAX8, whereas these markers are generally absent in metastatic lesions (26). In contrast, GATA3, ER, PR, HER2, and SOX10 are important markers for identifying breast cancer (27). In the present case, diffuse thyroid changes were observed, and FNAB was performed on the nodule in the inferior pole of the right thyroid lobe and on the right lateral neck lymph nodes. In the absences of IHC, the pathology department initially diagnosed the FNAB specimen as primary thyroid carcinoma, due to the morphological similarity between invasive ductal carcinoma of the breast and primary thyroid tumors such as follicular neoplasms (2, 15, 28). This diagnostic discrepancy highlights the limitations of relying solely on morphology, as it may lead to inappropriate clinical decisions and delay in accurate diagnosis and treatment. Therefore, incorporation of IHC is essential for determining the primary tumor origin, particularly in patients with a history of malignancy presenting with atypical thyroid lesions (28).

In most cases of thyroid metastases from breast cancer, the thyroid is one of multiple metastatic sites. Accordingly, these patients are often in an advanced stage of disease, with poor prognosis and limited survival. Optimal treatment of thyroid metastases should be individualized, taking into account the biological characteristics of the primary tumor, the extent of metastatic disease, the patient’s surgical tolerance, and overall life expectancy. Multidisciplinary treatment is essential in this context. Surgical resection of thyroid metastases may relieve symptoms caused by compression of adjacent organs (9, 25). Thyroidectomy is recommended in selected patients with isolated thyroid metastasis and expected long-term survival (9, 13), whereas patients with widespread disease should be managed with systemic therapy, such as chemotherapy or other advanced treatment (21). In the present case, the patient was initially diagnosed with right thyroid papillary carcinoma involving the lateral lymph node (cT1N1bM0) and underwent total thyroidectomy, bilateral central neck dissection (Level VI), and right radical neck dissection (Level II, III, IV and Vb) in accordance with NCCN guidelines (29). Postoperative pathology, however, confirmed that the thyroid lesion represented metastatic breast cancer. Given that PET/CT demonstrated an oligometastatic state, we considered surgery to have provided both symptomatic benefit and a foundation for subsequent systemic therapy. Pathological findings further suggested secondary endocrine resistance (30). As the patient was premenopausal, first-line endocrine therapy was recommended according to NCCN and ABC5 guidelines, consisting of OFS with an AI and a CDK4/6i (30, 31). She is currently receiving goserelin (3.75mg subcutaneously every 4 weeks), letrozole (2.5mg orally once daily) and ribociclib (600mg orally once daily, days1–21 of a 28-day cycle), and remains free of recurrence to date.

## 4 Conclusion

In summary, we report a rare case of breast cancer with thyroid metastasis occurring after the diagnosis with Graves’ disease. This case suggests that thyroid dysfunction in breast cancer patients may be associated with underlying thyroid metastasis and therefore warrants careful clinical attention. Although the thyroid is an uncommon site of breast cancer metastasis, routine surveillance should include assessment of thyroid abnormalities, and periodic PET/CT may be useful to evaluate systemic disease status. When thyroid lesions are identified, histopathological confirmation is essential to guide appropriate treatment planning, with the goal of improving quality of life and potentially prolonging survival.

## Data Availability

The original contributions presented in the study are included in the article/supplementary material, Further inquiries can be directed to the corresponding author.
